# A case of type 1 diabetes mellitus with which localized insulin allergy was markedly alleviated by switching to insulin glulisine

**DOI:** 10.1007/s00592-016-0841-5

**Published:** 2016-02-02

**Authors:** Kahori Watanabe, Yoshiki Kusunoki, Tomoyuki Katsuno, Rie Nakae, Toshihiro Matsuo, Fumihiro Ochi, Masaru Tokuda, Takafumi Akagami, Masayuki Miuchi, Jun-ichiro Miyagawa, Mitsuyoshi Namba

**Affiliations:** 1 Division of Diabetes, Endocrinology and Metabolism, Department of Internal Medicine, Hyogo College of Medicine, 1-1, Mukogawa-cho, Nishinomiya, Hyogo 663-8501 Japan; 2 Division of Innovative Diabetes Treatment, Department of Internal Medicine, Hyogo College of Medicine, 1-1, Mukogawa-cho, Nishinomiya, Hyogo 663-8501 Japan

**Keywords:** Insulin glulisine, Insulin allergy, Skin allergological test, Continuous subcutaneous insulin infusion

## Introduction

There have been numerous reports of allergies with causes such as differences in the amino acid sequences between human insulin and insulin extracted from porcine and bovine pancreas. Insulin allergy leads to deterioration of blood glucose control and decrease in quality of life. However, the availability of genetically recombinant human insulin formulations has led to insulin allergy becoming uncommon [1], and such allergies also occur much more rarely when insulin analog formulations are administered.

The present report is about a patient who showed allergic reactions to various insulin formulations, but showed only a weak allergic reaction to insulin glulisine, so the symptoms of insulin allergy ceased when treatment was switched to insulin glulisine.

## Case presentation

The patient was a 28-year-old female with type 1 diabetes mellitus. In February 2011, she developed the subjective symptoms of oral dryness, polydipsia, and polyuria, although she did not consult a physician. In March 2011, her mother found her unconsciousness in her home, and she was taken as an emergency to a different hospital, where diabetic ketoacidosis and type 1 diabetes mellitus were diagnosed. Intensive insulin therapy was then initiated, using insulin aspart and insulin detemir. However, because of the instability of fluctuations in blood glucose level, the attending physician at that hospital recommended initiation of continuous subcutaneous insulin infusion (CSII), the patient was therefore referred to our hospital in April 2011, and CSII was initiated. She then continued to visit our hospital as an outpatient, and the hemoglobin A1c (HbA1c) level was maintained at approximately 6.5 % (48 mmol/mol) by means of CSII using insulin lispro.

The patient had told only a few close friends about having type 1 diabetes mellitus, so, when she travelled with other friends, and work colleagues, she chose to control her blood glucose by multiple daily injections rather than CSII. In addition, she had expressed strong interest in different insulin formulations, and various formulations, including insulin glulisine, had therefore been tried. As basal insulin, she had a history of use of insulin detemir and insulin glargine. She had not previously had particular problems with any of these formulations.

**Fig. 1:**
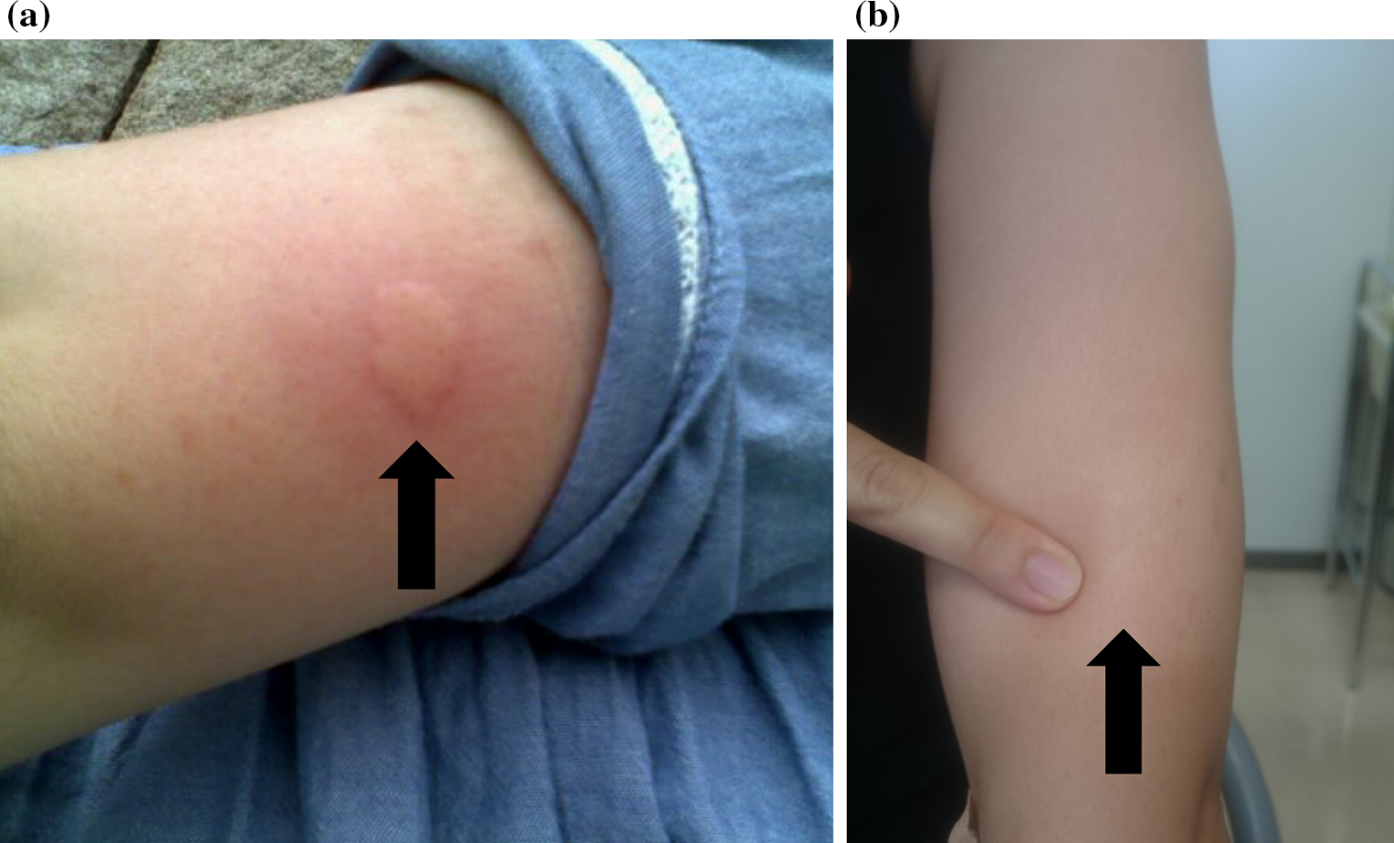
**a** Rash occurring when insulin lispro is administered. Erythema and swelling were found at the CSII cannula insertion site on the upper arm. **b** Five days after switching to insulin glulisine. No rash was found after switching to insulin glulisine

Since June 2015, the patient has developed the subjective symptoms of redness, swelling, and itchiness around the CSII cannula insertion site, and she was examined at our hospital in July. At that time, her blood glucose level was controlled by CSII with insulin lispro. At the time of examination, the casual blood glucose level was 157 mg/dL, the HbA1c level was 6.3 % (45 mmol/mol), and the body mass index was 21.8 kg/m^2^. Examination showed erythema and induration at the cannula insertion site, so localized insulin allergy was suspected (Fig. 1a). Therefore, the IgE antibody specific to human insulin was measured, the concentration was found to be 2.32 UA/mL (normal range ≤ 0.34 UA/mL), and the condition was judged to be class 2 (positive). The concentration of nonspecific IgE antibodies, on the other hand, was 21.8 IU/mL with no elevation (normal range ≤ 173 IU/mL), and no eosinophilia was found (white blood cell count: 6410 cells/μL; eosinophil percentage of white blood cell count: 0.6 %).

Next, intradermal tests were carried using various insulin formulations. Each insulin formulation was diluted to 0.05 units per 0.05 mL with physiological saline solution, and 0.02 mL of the resulting solution was injected intradermally, followed by evaluation. The findings were that mild erythema measuring 5 × 5 mm developed 15 min after intradermal injection of insulin glulisine, whereas with other insulin formulations redness developed immediately after intradermal injection, and erythema at least 10 mm in diameter and induration at least 5 mm in diameter developed after 15 min (Table 1). All rashes cleared up within 24 h after the intradermal test.

**Table 1:** Intradermal test results and additives in each insulin formulation

	Additive						Intradermal test
	Protamine sulfate	Zinc	Phenol	Cresol	Glycerin	Disodium hydrogen phosphate	Erythema (mm × mm)
Saline (control)							No reaction
Regular insulin (Humulin^®^ R)				○	○		17 × 17
NPH (Humulin^®^ N)	○	○	○	○	○	○	15 × 15
Aspart (NovoRapid^®^)		○	○	○	○	○	12 × 15
Glulisine (Apidra^®^)				○			5 × 5
Lispro (Humalog^®^)		○		○	○	○	14 × 20
Glargine (Lantus^®^)		○		○	○		12 × 13
Detemir (Levemir^®^)		○	○	○	○	○	10 × 12

On the basis of the above findings, localized insulin allergy was suspected; so the cannula insertion site was changed, and a basal insulin rate of CSII was maintained at 0.05–0.10 units/h, but no improvement of the rash was found. It was expected that reduction in this patient’s long-term basal insulin rate would result in severe hyperglycemia and/or ketosis, so the insulin formulation administered by CSII was switched from insulin lispro to insulin glulisine, with which the allergic reaction is milder, and no allergic symptoms such as redness and swelling were subsequently found at the cannula insertion site (Fig. 1b). In addition, the level of IgE antibody specific to human insulin was measured 8 weeks after the switch to insulin glulisine and was found to have decreased from 2.32 to 0.72 UA/mL.

## Discussion

In relation to allergic reactions to insulin formulations, it is essential to discriminate between reactions due to insulin and due to additives. Insulin is not manufactured in Japan, and it is not possible to order the additives for each insulin formulation separately. There have been reports that, in the case of patients with zinc allergies, the allergic symptoms cease when the formulation is switched to one that does not contain zinc, such as human insulin or insulin glulisine [2]. However, in the intradermal tests with the present patient, the result was positive even when humulin^®^ R which does not contain zinc as an additive was used, and a relationship between zinc and the allergic reaction could therefore be ruled out. Only sodium chloride is included as an isotonizing agent in insulin glulisine, whereas glycerin is used in other insulin formulations. In order to rule it out as an allergen, an intradermal test was carried out with diluted glycerin, and the finding was negative. For this reason, and also because of the positive result for an IgE antibody specific to human insulin, the allergy was indicated to be caused by insulin.

Desensitization by means of CSII has been reported to be useful for treating insulin allergy [3, 4]. There have been various reports about insulin levels at the time of initiation of desensitization, but, in general, the method used involves initiation at the minimum dose, followed by gradual dose increase, with monitoring of allergic symptoms. For example, in the case of type 2 diabetes mellitus, which is noninsulin dependent, the regimen that has been reported to be useful involves initiating basal insulin injection at 0.01 U/h, followed by gradual dose increase. However, in the case of type 1 diabetes mellitus, which is insulin-depleted, a regimen that involves initiation of insulin administration at very low dose, before increasing the dose, is difficult, because of risks of severe hyperglycemia and diabetic ketoacidosis. In the case of the present patient, administration was initiated at 0.05 U/h, in order to avoid hyperglycemia and ketoacidosis, but no alleviation of allergic symptoms was found. Therefore, the treatment was switched, after obtaining the patient’s consent, to insulin glulisine, this being the formulation with which the weakest reaction had been found in the intradermal tests, and the allergic symptoms disappeared as a result.

The reasons why the allergic reaction is alleviated when insulin glulisine is used may be suggested as follows. Human insulin readily forms hexamers, which delays its absorption from a subcutaneous site. The amino acids at positions 28–29 on the insulin molecule are the cause of the hexamerization. In insulin aspart, the proline at position 28 on chain B is substituted with aspartic acid, and in insulin lispro, the proline at position 28 on chain B is substituted with lysine, and the lysine at position 29 on chain B is substituted with proline, resulting in inhibition of hexamerization by both molecules. In insulin glulisine, the asparagine at position 3 on chain B of human insulin is substituted with lysine, and the lysine at position 29 on chain B is substituted with glutamic acid, as a result of which the only aggregate formation that takes place is conversion of monomers to dimers, with conversion of dimers to hexamers being inhibited [5]. In practice, insulin glulisine undergoes self-association, converting monomers to dimers and hexamers, less readily than other insulin formulations, suggesting that most of the insulin glulisine present in the formulation is in the monomeric form [5]. As the monomer has a lower molecular weight than the dimer and hexamer, it is probable that insulin glulisine, in which the monomer proportion is relatively high, has lower antigenicity than other formulations. In addition, as much of insulin glulisine is in the monomeric form, it is probable that it is transferred from the tissues to the bloodstream more rapidly than with other formulations, which means that it will give rise to local allergic symptoms less readily.

There have been reports of decreased IgE antibody titer, associated with alleviation of allergic symptoms, when patients with insulin allergies are treated by desensitization. In the case of the present patient, the IgE antibody specific to human insulin titer decreased, not with desensitization, but when treatment was switched to insulin glulisine administration. The following are considered to be the reasons for this patient’s decrease in IgE antibody titer: (1) The proportion of monomer in insulin glulisine is higher than in other insulin formulations, and the potential for antigenicity is therefore lower. (2) The time for which insulin remains in the subcutaneous position is less than with other insulin formulations. However, various factors have complicated relationships with the immune response and development of allergic symptoms, and further investigation is therefore needed.

## Conclusions

When insulin allergy is present, cross-antigenicity with numerous insulin formulations occurs, as in the case of the present patient. As insulin glulisine is mostly in the monomeric form, and is transferred to the bloodstream rapidly, it is probable that it has less tendency to cause insulin allergy.
